# Cigarette smoke exposure induces ROS-mediated autophagy by regulating sestrin, AMPK, and mTOR level in mice

**DOI:** 10.1080/13510002.2019.1601448

**Published:** 2019-04-06

**Authors:** Ana Lucia Bernardo Carvalho Morsch, Elvis Wisniewski, Thais Fernandes Luciano, Vitor Hugo Comin, Gustavo de Bem Silveira, Scherolin de Oliveira Marques, Anand Thirupathi, Paulo Cesar Silveira Lock, Claudio Teodoro De Souza

**Affiliations:** a Laboratory of Exercise Biochemistry and Physiology, Graduate Program in Health Sciences, Extremo Sul Catarinense University, Criciúma, Brazil; b Laboratory of Molecular Iron Metabolism, The Key Laboratory of Animal Physiology, Biochemistry and Molecular Biology of Hebei Province, College of Life Science, Hebei Normal University, Shijiazhuang, People’s Republic of China; c Department of Internal Medicine, Medicine School, Federal University of Juiz de Fora, Juiz de Fora, Brazil

**Keywords:** Autophagy, sestrin, smoke cigarette, lung, oxidative stress

## Abstract

Many pathological conditions linked to cigarette smoking are caused by the production of reactive oxygen species (ROS). The present study was conducted to analyze the effect of ROS on the lungs of Swiss mice exposed to cigarette smoking, focusing on autophagy-mediated mechanisms, and investigate the involvement of SESN2, AMPK, and mTOR signaling. Mice were exposed to cigarette smoke (CS) for 7, 15, 30, 45, and 60 days; the control group was not exposed to CS. Only mice exposed to CS for 45 days were selected for subsequent *N*-acetylcysteine (NAC) supplementation and smoke cessation analyses. Exposure to CS increased the production of ROS and induced molecular changes in the autophagy pathway, including an increase in phosphorylated AMPK and ULK1, reduction in phosphorylated mTOR, and increases in SESN2, ATG12, and LC3B levels. NAC supplementation reduced ROS levels and reversed all molecular changes observed upon CS treatment, suggesting the involvement of oxidative stress in inducing autophagy upon CS exposure. When exposure to CS was stopped, there were decreases in the levels of oxidative stress, AMPK and ULK1 phosphorylation, and autophagy-initiating molecules and increase in mTOR phosphorylation. In conclusion, these results suggest the involvement of ROS, SESN2, AMPK, and mTOR in the CS-induced autophagic process in the lung.

## Introduction

The human airway system is continuously exposed to numerous chemical toxicants, which lead to pulmonary exacerbations. Long-term exposure to cigarette smoke increases the risk of pulmonary-related diseases [1]. In response to irritant exposure, reactive oxygen species (ROS) activation, inflammatory response, and apoptosis occur, damaging the lung alveoli and leading to pulmonary pathologies, including chronic obstructive pulmonary disease (COPD)-emphysema. COPD-emphysema is a major leading cause of chronic morbidity and mortality worldwide and is projected to become the third leading cause of death in 2020 [2]. Clinically, alveolar emphysema is a primary cause of COPD and thus its early diagnosis may contribute to controlling this pathological condition. Recent evidence supports an important role for autophagy in the progression of COPD-emphysema [1,3,4]. Autophagy is an intracellular mechanism by which damaged cellular components and proteins are degraded and recycled by the cell itself as a defensive mechanism.

Cigarette smoke (CS) exposure induces the autophagy process to maintain cellular homeostasis. Additionally, autophagy flux may contribute to increasing epithelial cell loss. Although numerous studies have evaluated CS-induced autophagy, the importance of autophagy components in CS-linked oxidative stress conditions remains unclear. Autophagy components including autophagy-related protein (ATG) family kinase, Unc-51-like autophagy activating kinase (ULK1), and ULK2 in mammals are key regulators of autophagy initiation. The effect of the cigarette smoking on these components in the lungs is not well-understood. Additionally, the molecular basis of the inhibitory function of mammalian target of rapamycin (mTOR) in lung autophagy regulation is poorly understood. AMP-activated protein kinase (AMPK) is another strong candidate for influencing autophagy function and acts as an energy sensor to regulate cellular homeostasis [5]. AMPK induces autophagy in response to different cellular stresses, including oxidative stress [5–8]. Although the molecular mechanism underlying how ROS modulates AMPK has not been fully established, a role for the sestrin (SESN) family has been proposed [9].

SESN2 belongs to the family of highly conserved antioxidant proteins. Although it does not possess intrinsic catalytic antioxidant activity, SESN2 plays an important role in suppressing ROS [10]. As a family of stress-inducible proteins, SESNs have been reported to be up-regulated and activated upon exposure to DNA damage, oxidative stress, and hypoxia [9]. There are three isoforms, including Sestrin1 (Sesn1), Sestrin2 (Sesn2), and Sestrin3 (Sesn3). In mammals, SESN2 is thought to reduce oxidative stress by activating the nuclear factor erythroid 2-related factor 2 (NRF2) transcription factor [10,11]. Furthermore, it is well-documented that SESNs are involved in regulating mTOR signaling by activating AMPK [8,9,12,13]. Therefore, we hypothesized that ROS play a major role in the initiation of autophagy in response to cigarette smoking. Additionally, this study was conducted to evaluate the effects of ROS on SESN, AMPK, and molecules involved in autophagy in a CS exposure model.

## Materials and methods

### Animals

Two-month-old Swiss male mice were used for the experiments. All experimental procedures were performed in accordance with the Brazilian Guidelines for the Care and Use of Animals for scientific and didactic purposes and local ethics committee of the Extremo Sul Catarinense University (protocol number – 016/2013). A standard diet (Nuvilab CR1, Nuvital Nutrientes S/A, Brazil) and water were provided *ad libitum*. The animals were maintained at 70% humidity and a temperature of 20 ± 2°C, with a 12 h light–dark cycle. The mice were periodically checked to verify their pathogen-free status. The study was divided into three stages.

### Experimental design

#### Stage 1 – CS treatment for different exposure times

Sixty animals were randomly divided into six groups (*n* = 10) and treated with CS for 7, 15, 30, 45, and 60 days; non-exposed animals were used as a control and were evaluated for 60 days. After the exposure period, fragments of the right lung were collected for western blotting and dichlorofluorescein (DCFH) analysis. SESN2, AMPK, and mTOR expression levels were measured. Each time the animals were exposed to CS, the control group (in all stages) was placed in the laboratory without exposure to CS.

#### Stage 2 – supplementation with the antioxidant NAC in mice exposure to cigarette smoke for 45 days

To investigate the involvement of ROS in activating autophagy, mice exposed to CS for 45 days were selected for *N*-acetylcysteine (NAC) administration. The selection of 45 days of CS exposure for NAC supplementation was based on the levels of SESN2, AMPK, and mTOR obtained from stage 1 results. We used NAC supplementation to counteract the ROS involvement in activating the autophagy process. Briefly, 40 mice were randomly divided into four groups (*n* = 10): Cont (non-CS exposed); Cont + NAC (non-CS exposed + NAC supplementation); 45 (CS exposure for 45 days); and 45 + NAC (45 days of CS exposure + NAC supplementation). The antioxidant NAC (daily single dose of 60 mg/kg, in 0.5 mL) was administered orally every day by oral gavage using a cannula as previously described [13]. After the exposure period, fragments of the right lung were collected for western blotting and DCFH analysis.

#### Stage 3 – effect of cessation of CS exposure on ROS-mediated autophagy

The involvement of ROS in initiating autophagy was evaluated by performing cessation of exposure to CS for different periods after 45 days of CS exposure. Briefly, 60 animals were randomly divided into six groups (*n* = 10): Cont (non-CS-exposed); 45 CS (45 CS exposure days); 45 CS exposure days + 7 days of cessation; 45 CS exposure days + 15 days of cessation; 45 CS exposure days + 30 days of cessation; and 45 CS exposure days + 45 days of cessation. After treatment, fragments of the right lung were collected for western blotting and DCFH analysis.

### CS exposure

Mice were exposed every day to 12 commercially available filtered cigarettes containing a total of 8 mg of tar and 0.6 mg of nicotine as described previously [14,15]. Briefly, the mice were placed in an inhalation chamber (40 cm long, 30 cm wide, and 25 cm high) inside an exhaustion chapel. One cigarette was inserted into a 60-mL syringe and 20 smoke puffs of 50 mL each were aspirated with the syringe and then immediately pushed into the chamber to reach a total of 1 L of smoke per cigarette. The animals were maintained under 3% smoke–air conditions for 6 min. Next, the cover was removed from the inhalation chamber; by turning on the exhaust fan of the chapel, the smoke was evacuated within 1 min. The mice were then immediately exposed to CS from a second cigarette for 6 min. This exposure protocol was repeated for four cigarettes, three times per day (morning – 8:30 AM, noon – 13:30 PM, and afternoon – 17:30 PM), resulting in 72 min of smoke exposure per day. The average concentration of carbon monoxide within the chamber ranged from 499 ppm to 732 ppm during the exposure period (Instrutherm LTDA, São Paulo, Brazil). The mice were maintained under this smoke air condition (8 ± 24 mg tar, 0.6 ± 0.01 mg nicotine) for 6 min in three daily sessions [14]. At 12 h after the last CS exposure, the animals were sacrificed by decapitation. Fragments of lung tissue were homogenized in buffer solution for further analysis.

### Quantification of ROS by DCFH diacetate (DCFH-DA)

ROS were detected using the DCFH-DA probe as previously described [16]. Briefly, lung tissue was homogenized and then DCFH-DA was added to the sample and incubated for 30 min at 37°C. Free radicals in the samples caused oxidation of DCFH, resulting in release of a fluorescent product, which can be readily measured using a fluorimeter (488 nm emission and 525 nm excitation) [17]. In this assay, 100 µL of water and 75 µL of DCFH-DA were added to 25 µL of sample homogenate. The homogenates were vortexed and placed in a 37°C water bath under a light for a period of 30 min. The calibration curve was prepared using 0.1 µM DCF diluted to different concentrations in phosphate/EDTA buffer at pH 7.4 as a standard. Both the samples and standard were processed in duplicate in the dark. After 30 min, the readings were measured on the fluorimeter (488 nm emission and 525 nm excitation) [17]. The results are expressed as mmol per mg of proteins.

### SDS-PAGE and western blotting

Lung tissue was collected and immediately homogenized in lysis buffer (1% Triton-X 100, 100 mM Tris, pH 7.4, containing 100 mM sodium pyrophosphate, 100 mM sodium fluoride, 10 mM EDTA, 10 mM sodium vanadate, 2 mM PMSF, and 0.1 mg of aprotinin/mL) at 4°C with a Polytron MR 2100 (Kinematica, Luzern, Switzerland). The lysate was centrifuged at 12,851 × *g* for 40 min at 4°C (Eppendorf AG 5804R, Hamburg, Germany) to remove the debris, and the supernatant was used for protein quantification using the Lowry method [18]. A total of 105 µg of total protein was denatured by boiling in Laemmli buffer containing 100 mM DTT, separated by 10% sodium dodecyl sulfate-polyacrylamide gel electrophoresis under reducing conditions, and then transferred to a nitrocellulose membrane. The membrane was blocked, probed, and developed as described previously [19]. Antibodies used for immunoblotting were phospho AMPK^thr172^, phospho mTOR^ser2448^, phospho ULK1^ser317^, Atg12 (Cell Signaling Technology, Beverly, MA, USA) and Sestrin 2, LC3B (Santa Cruz Biotechnology, Dallas, TX, USA). The membranes were then incubated with peroxidase-conjugated secondary antibody for 2 h at room temperature. Next, the membranes were incubated for 2 min with enzymatic substrate and exposed to the RX film on a radiographic development cassette. The intensity and area of the bands were captured using a scanner (HP G2710), quantified using the Scion Image program (Scion Corporation, Frederick, MD, USA), and expressed as arbitrary units. After blotting, the membranes were probed again with α-tubulin antibody to serve as the protein loading control.

### Statistical analysis

The data were expressed as the mean and standard error of means (mean ± SEM) and analyzed statistically by one-way analysis of variance (ANOVA), followed by Tukey’s post-hoc test. The level of significance was set to 95% (*p* < 0.05). SPSS version 18.0 for Windows (SPSS, Inc., Chicago, IL, USA) was used for data analysis.

## Results

**Figure 1.**
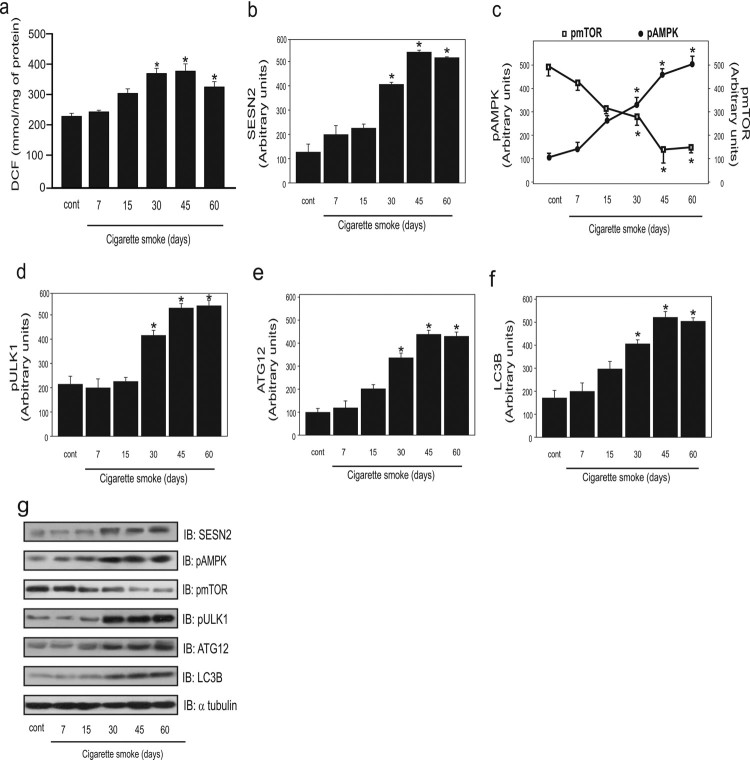
Effect of cigarette smoke (CS) on levels of ROS and autophagy markers in the lungs of mice exposed to CS for different durations (*n* = 10 per group). Dichlorofluorescein (DCF) level (a), SESN2 protein levels (b), AMPK and mTOR phosphorylation (c), ULK1 phosphorylation (d), ATG12 protein levels (e), LC3B protein levels (f), and representative western blot analysis of the evaluated proteins (g) in control (non-exposed group) and for different days of CS exposure (7, 15, 30, 45, and 60 days). Data are expressed as the mean and standard error of the means (mean ± SEM) and analyzed statistically by one-way analysis of variance, followed by Tukey’s HSD post hoc test (*p < 0.05 versus control).

In the present study, we analyzed the involvement of ROS in CS autophagy. Based on a previous study, we chose 7, 15, 30, 45, and 60 days of exposure to attain high levels of ROS [14]. We found that the oxidation of DCFH (DCF) was greater at 30, 45, and 60 days of exposure when compared to the group not exposed to cigarette smoke and in the 7 days of CS exposure group (Figure 1A). Next, we analyzed whether ROS modulated the levels of autophagic molecules. First, we analyzed the protein levels of SESN2. A significant increase in the protein levels of SESN2 at 30, 45, and 60 days of CS exposure was observed compared to the control group (Figure 1B).

Previous studies showed that SESN2 regulates AMPK [9], and thus we analyzed the phosphorylation level of this molecule. AMPK phosphorylation was significantly higher at 30, 45, and 60 days of CS exposure. In contrast, mTOR phosphorylation was lower at 30, 45, and 60 days, revealing an inverse relationship between these two molecules (Figure 1C). A significant increase in ULK1 phosphorylation was observed at 30, 45, and 60 days of CS exposure compared to that in the control group (Figure 1D). Further, the protein level of ATG12 was significantly increased at 30, 45, and 60 days compared to that in the control group (Figure 1E). Additionally, increased protein levels of LC3B were observed at 30, 45, and 60 days of CS exposure compared to that in the control and animals exposed to CS for 7 days (Figure 1F).

**Figure 2.**
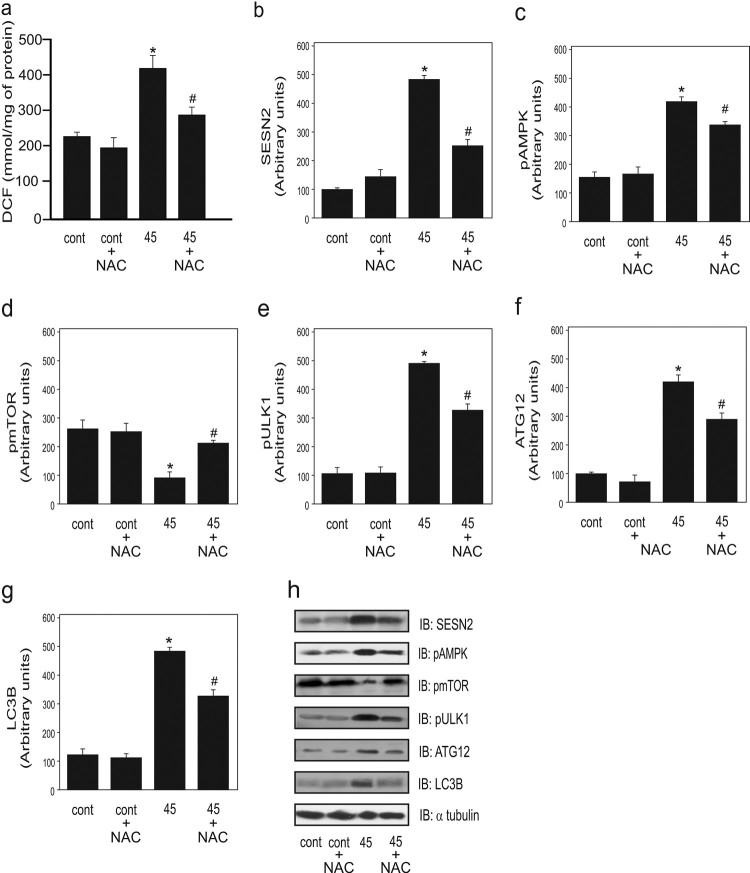
Effect of antioxidant NAC supplementation on ROS and autophagy markers in the lungs of mice exposed to cigarette smoke (CS) for 45 days (*n* = 10 per group). Dichlorofluorescein (DCF) level (a), protein levels of SESN2 (b), AMPK phosphorylation (c), mTOR phosphorylation (d), ULK1 phosphorylation (e), ATG12 (f), LC3B (g), and representative western blot of the considered proteins (h) in the control (non-exposed group), control plus NAC, CS exposure for 45 days without NAC, and 45 days of CS exposure with NAC. Data are expressed as the mean and standard error of the means (mean ± SEM) and analyzed statistically by one-way analysis of variance, followed by Tukey’s HSD post hoc test. (*p < 0.05 versus control and control + NAC; ^#^*p* < 0.05 versus 45 + NAC).

Next, we evaluated the effect NAC supplementation on the parameters described above. We observed that the ROS level was decreased following supplementation with NAC as compared to at 45 days of CS exposure (Figure 2A). Additionally, the SESN2 level was significantly decreased after supplementation with NAC compared to that in the non-supplemented group, confirming that oxidative stress is involved in in regulating the protein levels of SESN2 (Figure 2B). Moreover, there was a significant reduction in the phosphorylation of AMPK in the NAC-supplemented group compared to that in the non-supplemented group (Figure 2C). In contrast, a significant increase in mTOR phosphorylation was observed following NAC supplementation compared to that in the CS-exposed group without supplementation (Figure 2D). Further, there was a significant decrease in ULK1 phosphorylation following NAC supplementation compared to that in the non-supplemented group (Figure 2E). For autophagy components, a significant decrease in the protein levels of ATG12 and LC3B was observed in the group exposed to CS for 45 days with NAC supplementation compared to that in the non-supplemented 45 days group (Figure 2F and 2G respectively).

**Figure 3.**
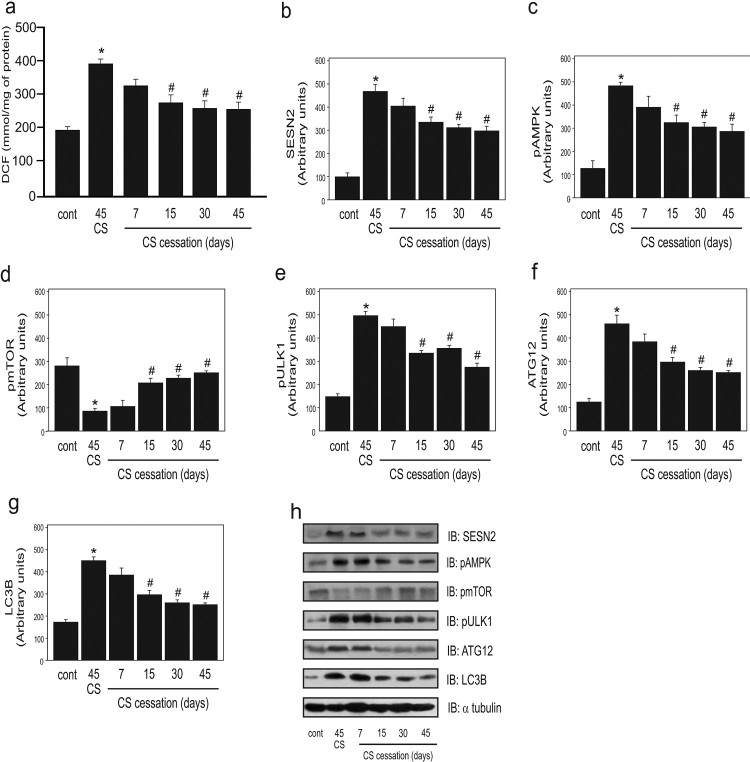
Effect of cigarette smoke (CS) cessation on ROS and autophagy markers in the lungs of mice after 45 days of CS exposure (*n* = 10 per group)*.* Dichlorofluorescein (DCF) level (a), protein levels of SESN2 (b), AMPK phosphorylation (c), mTOR phosphorylation (d), ULK1 phosphorylation (e), ATG12 (f), LC3B (g), and representative western blot analysis of the evaluated proteins (h) in the control (non-exposed group), CS exposure for 45 days, and 45 days CS exposure followed by CS cessation for different days (7, 15, 30, and 45 days) groups. Data are expressed as the mean and standard error of the means (mean ± SEM) and analyzed statistically by one-way analysis of variance, followed by Tukey’s HSD post hoc test (*p < 0.05 versus cont; ^#^*p* < 0.05 versus 45 CS).

In step III, we hypothesized that if CS increased ROS, leading to the augmentation of autophagy, the cessation of CS exposure would reduce the autophagic process. Therefore, we subjected the animals to 45 days of CS exposure and subsequent cessation at different times, (7, 15, 30, and 45 days). A significant reduction in DCF levels at 15, 30, and 45 days of cessation was observed as compared to CS exposure for 45 days group (Figure 3A). Additionally, there was a significant decrease in the SESN2 protein levels and AMPK phosphorylation at 15, 30, and 45 days of cessation compared to CS exposure for 45 days group (Figure 3B and 3C). In contrast, mTOR phosphorylation showed a significant and progressive increase at 15, 30, and 45 days of cessation compared to CS exposure for 45 days group. Furthermore, a significant decrease was observed in ULK1 phosphorylation following cessation at 15, 30, and 45 days (Figure 3D and 3E). We also observed a significant reduction in the levels of the autophagy proteins ATG12 and LC3B at 15, 30, and 45 days of cessation (Figure 2F and 2G).

## Discussion

ROS-induced autophagy in response to CS in COPD is not well-understood. CS induces pro-oxidants to increase oxidative stress in epithelial cells and other cells; autophagy is involved in this mechanism. The present study demonstrated the effect of CS on molecules involved in autophagy. We first established the time of CS exposure for ROS to reach high levels. Considering that the lungs are the primary targets and are susceptible to oxidative damage caused by smoking, ROS levels were evaluated in the lung tissue after different exposure times. The present study revealed a significant increase in DCFH oxidation at 30, 45, and 60 days of CS exposure. A study by Raza and colleagues [20] exposed BALB/C mice to nine cigarettes daily for 4 days and observed an increase in ROS (as measured by DCFH) in the lung tissue as compared to in controls. Additionally, a similar study conducted by Carlos et al. [21] using C57BL/6 mice reported a significant increase in ROS after exposure to CS.

To verify the changes in the cellular redox status and autophagy induced by an antioxidant, the mice were treated with NAC. DCF levels were significantly reduced at 45 days of CS exposure + NAC as compared to at 45 days CS exposure without NAC. This result was expected, as NAC is a powerful thiolic molecule with antioxidant properties. NAC exerts antioxidant activity directly through its three sulfhydryl groups, which provide electrons to neutralize free radicals, and indirectly by replenishing the intracellular levels of glutathione [22]. In the respiratory system, antioxidant protection is mainly mediated by glutathione present in the epithelial tissue by converting from the reduced form to the oxidized form, after being converted again to the form reduced by the enzyme glutathione reductase [23]. Similar results were found following the cessation of CS exposure. When CS exposure was stopped, the ROS level reduced at 15, 30, and 45 days and the levels of autophagy proteins were also reduced, demonstrating the involvement of ROS in the autophagic process. Further, the protein levels of SESN2 were significantly increased in the lung tissue at 30, 45, and 60 days of exposure to CS. A study by Heidler [24] conducted with SESN2 knockout mice exposed to CS for 6 h a day, 5 days a week for 8 months, showed that inactivation of SESN2 protected the animals from development of CS-induced pulmonary emphysema. Moreover, the same authors observed overexpression of SESN2 in the lungs of smokers and particularly in the lungs of individuals with advanced COPD compared to in healthy subjects.

After exposure to exacerbated stimuli, increased levels of SESN2 can activate AMPK, resulting in the negative regulation of mTOR [7,9,12]. In the present study, we also observed reduced SESN2 protein levels and AMPK phosphorylation and increased mTOR phosphorylation in the group exposed to CS for 45 days and supplemented with NAC. Kim and colleagues [25] treated the cells with quercetin and quercetin plus NAC and observed that, in cells treated with quercetin alone, there was an increase in apoptosis and ROS generation, which was responsible for the increase in SESN2 expression accompanied by activation of AMPK. In cells treated concomitantly with NAC, the authors detected decreases in SESN2, p53 expression, and AMPK phosphorylation, which induced mTOR activation. The researchers further showed that mTOR activity induced by SESN2 was dependent on the phosphorylation of AMPK [25]. In the present study, oxidative stress was reduced, which was confirmed by the decrease in DCF levels after 15 days of cessation of CS exposure and by a decrease in the protein levels of SESN2, demonstrating a relationship between oxidative stress and SESN2 levels. The present study showed that the inverse relationship between mTOR and AMPK was dependent on the time of exposure to CS. Additionally, we observed that increased expression of SESN2 was related to the phosphorylation of AMPK and decreased phosphorylation of mTOR. There was also a significant decrease in AMPK phosphorylation at 15, 30, and 45 days of cessation of CS exposure and, as expected, there was an increase in mTOR phosphorylation, which was significant at 15, 30, and 45 days of cessation.

Autophagy occurs when AMPK and mTOR interact through coordinated phosphorylation of ULK1 [5,26]. In the present study, increased phosphorylation of ULK1 was observed at 30, 45, and 60 days of CS exposure compared to that in the control group. ULK1 is a downstream enzyme in the mTOR signaling pathway that regulates phagophore membrane nucleation and initiates autophagosome formation, and its activation is crucial for the initiation of autophagy [27]. After elongation of the phagophore, a second complex LC3–PE forms, which is essential for allowing the process of sequestration of cellular components to be damaged [28]. LC3B has been analyzed in experimental studies as a marker of autophagosome formation [29]. In the present study, we observed significant elevation in the ATG12 and LC3B protein levels at 30, 45, and 60 days of CS exposure compared to in the control group. Zhu and colleagues treated epithelial cells in culture with CS extract [30] and observed a significant increase in the expression of LC3B-I and LC3B-II; they showed that this elevation in protein levels was dose- and time-dependent. A study by Chen et al. [31] revealed a change in autophagy as confirmed by increased levels of LC3B-II/I, ATG4, AT5, ATG12, and ATG7. Additionally, increased autophagosome formation was reflected as the observation of vacuoles by electron microscopy in the lungs of patients with COPD, unlike the small number of vacuoles detected in the tissues of the control group. These same authors knocked down LC3B in cultured human lung epithelial cells and observed that inhibition of autophagy protected the cells from CS exposure-induced apoptosis; further, in animals with genetic deficiency of LC3B, there was an association with the resistance to emphysema development following exposure to CS. In the present study, we observed decreased phosphorylation of ULK1 and protein levels of ATG12 at 15, 30, and 45 days and in the levels of LC3B at 7, 15, 30, and 45 days after cessation of exposure to CS, indicating a decrease in autophagy.

In conclusion, the present study revealed that exposure to CS led to an increase in ROS in the lung tissue along with increased SESN2 protein levels and phosphorylation of AMPK and decreased mTOR and ULK1 phosphorylation. Exposure to CS also led to increased levels of the autophagy marker molecules ATG12 and LC3B. These events were decreased by NAC supplementation and the cessation of exposure to CS. In future, detailed studies of this crucial mechanism of SESN2-dependent autophagy response will increase the understanding of the pathogenic effect of CS-associated chronic airway diseases.
